# 1-(2-Methyl-5-nitro­phenyl)guanidinium picrate

**DOI:** 10.1107/S1600536809037647

**Published:** 2009-10-17

**Authors:** Jerry P. Jasinski, Ray J. Butcher, M. T. Swamy, H. S. Yathirajan, A. R. Ramesha

**Affiliations:** a Department of Chemistry, Keene State College, 229 Main Street, Keene, NH 03435-2001, USA; b Department of Chemistry, Howard University, 525 College Street NW, Washington, DC 20059, USA; c Department of Studies in Chemistry, University of Mysore, Manasagangotri, Mysore 570 006, India; d RL Fine Chem, Bangalore 560 064, India

## Abstract

In the crystal structure of the title salt, C_8_H_11_N_4_O_2_^+^·C_6_H_2_N_3_O_7_^−^, the pictrate anion participates in extensive hydrogen bonding with the guanidinium ion group of the cation, linking the mol­ecules through N^+^—H⋯O^−^ hydrogen bonds and inter­molecular N—H⋯O and C—H⋯O inter­actions. These hydrogen-bonding configurations involve two three-centre/bifurcated bonds [N—H⋯(O,O)] that are observed between two N atoms from the guanidinium ion group of the cation and the *o*-NO_2_ and phenolate O atoms of the picrate anion. In addition, π–π inter­actions also contribute to the crystal packing, with a centroid-to-centroid distance of 3.693 (6) Å and a slippage angle of 1.614°. A significant number of conformational differences are observed between the salt in the crystal structure and the models obtained by density functional theory (DFT) calculations of the geometry-optimized structure.

## Related literature

For background literature, see: Berlinck (2002 ▶); Heys *et al.* (2000 ▶); Ishikawa & Isobe (2002 ▶); Kelley *et al.* (2001 ▶); Laeckmann *et al.* (2002 ▶); Moroni *et al.* (2001 ▶); Orner & Hamilton (2001 ▶); Zyss *et al.* (1993 ▶). For related structures, see: Cunningham *et al.* (1997 ▶); Demir *et al.* (2006 ▶); Gupta & Dutta (1975 ▶); Moghimi *et al.* (2005 ▶); Murtaza *et al.* (2007 ▶, 2009 ▶); Pereira Silva *et al.* (2007 ▶); Pruszynski *et al.* (1992 ▶); Ren *et al.* (2007 ▶); Sonar *et al.* (2007 ▶); Smith *et al.* (2007 ▶, 2007*a* ▶); Stanford *et al.* (2007 ▶); Stępień & Grabowski (1977 ▶); Wang *et al.* (2009 ▶); Wei (2008 ▶). For density functional theory (DFT), see: Becke (1988 ▶, 1993 ▶); Frisch *et al.* (2004 ▶); Hehre *et al.* (1986 ▶); Lee *et al.* (1988 ▶); Schmidt & Polik (2007 ▶). For the Cambridge Structural Database, see: Allen (2002 ▶); Bruno *et al.* (2004 ▶).
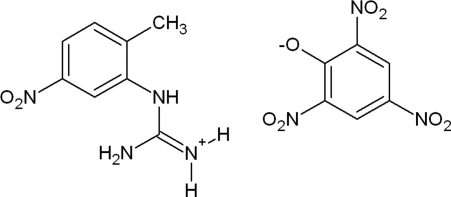


## Experimental

### 

#### Crystal data

C_8_H_11_N_4_O_2_^+^·C_6_H_2_N_3_O_7_^−^
*M* *_r_* = 423.31
Triclinic, 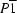

*a* = 7.1318 (10) Å
*b* = 10.6239 (13) Å
*c* = 11.9564 (13) Å
α = 84.257 (10)°
β = 74.497 (11)°
γ = 77.559 (11)°
*V* = 851.58 (18) Å^3^
*Z* = 2
Cu *K*α radiation
μ = 1.23 mm^−1^
*T* = 110 K
0.51 × 0.41 × 0.33 mm

#### Data collection

Oxford Xcalibur diffractometer with Ruby (Gemini Cu) detector
Absorption correction: multi-scan (*CrysAlis Pro*; Oxford Diffraction, 2009 ▶) *T*_min_ = 0.431, *T*_max_ = 1.000
6248 measured reflections
3344 independent reflections
2761 reflections with *I* > 2σ(*I*)
*R*_int_ = 0.021

#### Refinement

*R*[*F* ^2^ > 2σ(*F* ^2^)] = 0.041
*wR*(*F* ^2^) = 0.118
*S* = 1.07
3344 reflections
272 parameters
H-atom parameters constrained
Δρ_max_ = 0.27 e Å^−3^
Δρ_min_ = −0.29 e Å^−3^

### 

Data collection: *CrysAlis Pro* (Oxford Diffraction, 2009 ▶); cell refinement: *CrysAlis RED* (Oxford Diffraction, 2007 ▶); data reduction: *CrysAlis RED*; program(s) used to solve structure: *SHELXS97* (Sheldrick, 2008 ▶); program(s) used to refine structure: *SHELXL97* (Sheldrick, 2008 ▶); molecular graphics: *SHELXTL* (Sheldrick, 2008 ▶); software used to prepare material for publication: *SHELXTL*.

## Supplementary Material

Crystal structure: contains datablocks global, I. DOI: 10.1107/S1600536809037647/kp2228sup1.cif

Structure factors: contains datablocks I. DOI: 10.1107/S1600536809037647/kp2228Isup2.hkl

Additional supplementary materials:  crystallographic information; 3D view; checkCIF report

## Figures and Tables

**Table 1:** Hydrogen-bond geometry (Å, °)

*D*—H⋯*A*	*D*—H	H⋯*A*	*D*⋯*A*	*D*—H⋯*A*
N2*A*—H2*AA*⋯O22*B*^i^	0.88	2.20	3.0737 (18)	171
N3*A*—H3*AB*⋯O1*B*	0.88	2.03	2.7866 (18)	143
N3*A*—H3*AB*⋯O62*B*	0.88	2.35	3.0882 (18)	142
N3*A*—H3*AC*⋯O1*A*^ii^	0.88	2.32	2.9808 (19)	132
N4*A*—H4*AA*⋯O1*B*	0.88	1.98	2.7499 (18)	145
N4*A*—H4*AA*⋯O21*B*	0.88	2.34	3.0683 (19)	141
N4*A*—H4*AB*⋯O21*B*^i^	0.88	2.11	2.9572 (18)	163
C3*A*—H3*AA*⋯O41*B*^iii^	0.95	2.37	3.262 (2)	155
C7*A*—H7*AB*⋯O1*B*^iv^	0.98	2.56	3.447 (2)	151

Symmetry codes: (i) 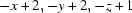; (ii) 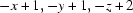; (iii) 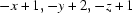; (iv) 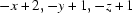.

**Fig. 1.**
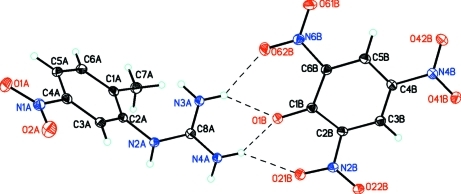
The asymmetric unit of (I), showing the bifrucated (three-centre) N—H···(O,O) donor hydrogen bond configuration and the atom labeling scheme. Hydrogen bonds are shown as dashed lines. Displacement ellipsoids are shown at the 50% probability level.

**Fig. 2.**
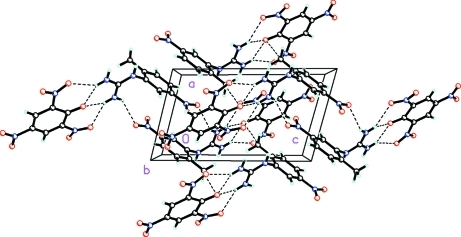
Packing diagram of the title compound, (I), viewed down the b axis. Dashed lines indicate intermolecular bifurcated (three-centre) N—H···(O,O), N—H···O, and C—H···O donor hydrogen bond interactions which produces a 2-D network arranged along the (101) plane.

**Fig. 3.**
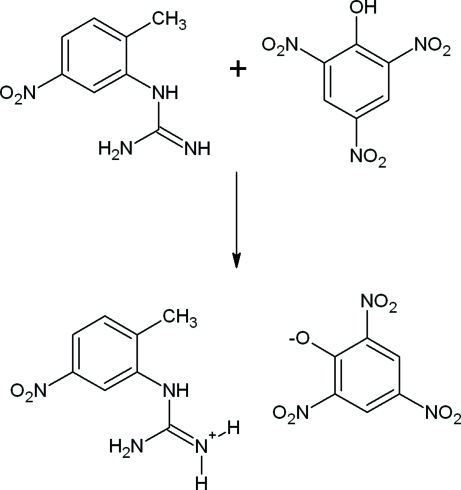
The formation of the title compound.

## supplementary crystallographic information

### Comment

Guanidines, important compounds that have many biological, chemical and medicinal applications (Berlinck, 2002; Heys *et al.*, 2000), have received increasing interest as medicinal agents with antitumour, antihypertensive, antiglaucoma and cardiotonic activities (Laeckmann *et al.*, 2002; Kelley *et al.*, 2001; Moroni *et al.*, 2001). Due to their strong basic character, they can be considered as super-bases that readily undergo protonation to generate resonance-stabilized guanidinium cations (Ishikawa & Isobe, 2002). Guanidine is used in variety of supramolecular recognition processes across the spectrum of organic, biological and medicinal chemistry (Orner & Hamilton, 2001) with special interest motivated by their potential applications in non-linear optics (Zyss *et al.*, 1993).

The crystal structures of a few related guanidine derivatives *viz.* guanidinium 2-amino-4-nitrobenzoate monohydrate at 130 K (Smith *et al.*, 2007), (2,4,6-trinitrophenyl)guanidine (Smith *et al.*, 2007*a*), 4-methoxy-phenylguanidinium chloride (Ren *et al.*, 2007), (*E*)-1-[(2-methoxyphenyl)methyleneamino]guanidinium chloride (Sonar *et al.*, 2007), have been reported. 1-(2-methyl-5-nitrophenyl)guanidine is one of the starting compounds for the synthesis of the anticancer drug, imatinib. In connection with the importance of guanidine derivatives, the present paper reports the crystal structure of the title compound, (I), C_14_H_13_N_7_O_9_.

The title compound, (I), crystallizes as a salt with one independent cation–anion pair [C_8_H_11_O_2_N_4_^+^.C_6_H_2_N_3_O_7_^-^] in the asymmetric unit (Fig. 1). Bond lengths and angles can be regarded as normal (Cambridge Structural Database, Version 5.30, February, 2009; Allen, 2002, *Mogul*, Version 1.1.3; Bruno *et al.*, 2004). In the cation, the angle between the dihedral planes of the guanidinium ion [(CH_5_N_3_)^+^] and the bonded 2-methyl-5-nitrophenyl group is 83.0 (7)°. All three nitrogen atoms (N2A, N3A & N4A) exhibit *sp*^2^ hybridization with a sum of angles around each atom of 360.0 (0)° resulting in a planar guanidinium ion group. The dihedral angle between the mean planes of the 5-nitro group and the benzyl ring is 5.0 (3)°. In the picrate anion, the mean plane of two *o*-NO_2_ and single *p*-NO_2_ groups are twisted by 14.6 (4)°, 16.6 (3)° and 5.6 (8)°, respectively, from the mean plane of the benzyl ring. The difference in the twist angles of the mean planes of the two *o*-NO_2_ groups can be attributed to an intermolecular hydrogen bonded interaction between the guanidinium ion of the cation with both of these groups (O21B—N2B—O22B & O61B—N6B—O62B) of the picrate anion, in which the O21B and O62B atoms form intermolecular "side" hydrogen bonds (N4A—H4AA···O21B & N3A—H3AB···O62B) with N4A and N3A from the guanidinium ion (Fig. 2, Table 1). N4A and N3A also both form intermolecular hydrogen bonds with the phenolate oxygen anion O1B (N4A—H4AA···O1B & N3A—H3AB···O1B), each creating a bifrucated (three-centre), N—H···(O,O) hydrogen bond. As a result, O1B and O21B act as the double acceptors. N4A and N3A form additional intermolecular hydrogen bonds with nearby O21B (N4A—H4AB···O21B) and O1A (N3A—H3AC···O1A) atoms, respectively. The dihedral angle between the mean planes of the benzyl ring and guanidinium ion group in the cation and the benzyl ring of the picrate anion are 80.8 (7)° and 7.4 (7)°, respectively. Additional weak N2A—H2AA···O22B and C3A—H3AA···O41B hydrogen bonds and π–π stacking interactions occur (*Cg*1···*Cg*1 = 3.693 (6) Å; -*x*, 1 - *y*, -*z*; slippage = 1.614°; *Cg*1 = C1A–C6A centroid) contributing to the stability of crystal packing.

A density functional theory (DFT) geometry optimization molecular orbital calculation (Schmidt & Polik, 2007) was performed on the C_8_H_11_O_2_N_4_^+^.C_6_H_2_N_3_O_7_^-^ cation–anion pair of the title molecule, (I), with the *GAUSSIAN03* program package (Frisch *et al.* 2004) employing the B3LYP (Becke three parameter Lee–Yang–Parr) exchange correlation functional, which combines the hybrid exchange functional of Becke (Becke, 1988, 1993) with the gradient-correlation functional of Lee, Yang and Parr (Lee *et al.* 1988) and the 3-21G basis set (Hehre *et al.* 1986). Starting geometries were taken from X-ray refinement data. The dihedral angle between the dihedral planes of the guanidinium ion [(CH_5_N_3_)^+^] and the bonded 2-methyl-5-nitrophenyl group in the cation decreases by 7.1 (2)° to 70.9 (1)° while the mean plane of the 5-nitro group decreases by 5.0 (3)° to become planar with the benzyl ring. In the picrate anion, the twist of the mean planes of two *o*-NO_2_ and single *p*-NO_2_ groups decreases by 12.3 (1)°, 10.4 (9)° and 5.6 (8)° to 4.3 (2)°, 4.1 (5)° and 0.0 (0)°, respectively, from the mean plane of the 6-membered benzyl ring. The dihedral angle between the mean planes of the benzyl ring and guanidinium ion group in the cation and the benzyl ring of the picrate anion change by -17.8 (5)° and +2.1 (9)° to 63.0 (2)° and 9.6 (6)°, respectively. Examination of the partial charges from the DFT geometry optimization indicate that H4AA (0.4.4384) is slightly more positive than H3A (0.402348) producing a slightly delocalized proton charge over the guanidium group and favoring the N4A atom. This coincides with a shorter N4A—H4AA···O1B (= 1.984 Å) hydrogen bond than N3A—H3A···O1B (= 2.033 Å) due to the multiple acceptor function of O1B. In conclusion, the significant number of conformational changes that are observed between the crystalline environment of this cation–anion (1-(2-methyl-5-nitrophenyl)guanidinium picrate) salt and that of a density functional theory calculation of the geometry optimized structure support the effects of strong intermolecular hydrogen bonding interactions and weak π–π ring intermolecular interactions between the guanidinium ion and bonded 2-methyl-5-nitrophenyl group of the cation and the *o*-NO_2_, *p*-NO_2_ and phenolate oxygen groups of the picrate anion as providing the major influence on packing effects in the crystal of the title compound, 1-(2-methyl-5-nitrophenyl)guanidinium picrate, (I).

### Experimental

The title compound was synthesized by adding a solution of picric acid (0.92 g, 2 mmol) in 10 ml of methanol to a solution of 1-(2-methyl-5-nitrophenyl)guanidine (0.45 g, 2 mmol) in 10 ml of methanol (Scheme 2). A yellow colour developed and the solution was allowed to evaporate slowly at room temperature.The yellow colour compound formed was filtered off, washed several times with diethyl ether, and then dried over CaCl_2_ (yield: 64.2%). Crystals for X-ray studies were grown by slow evaporation of dimethyl formamide solution. The melting range was found to be 382–385 K. Analysis found (calculated) for C_14_H_13_N_7_O_9_ (%): C: 39.95 (39.72), H: 2.99 (3.1), N: 23.36 (23.16).

### Refinement

All of the H atoms were placed in their calculated positions and then refined using the riding model with N—H = 0.88, C—H = 0.95 Å, and with *U*_iso_(H) = 1.17–1.51*U*_eq_(C,N).

### Crystal data

**Table:**

C_8_H_11_N_4_O_2_^+^·C_6_H_2_N_3_O_7_^−^	*Z* = 2
*M**_r_* = 423.31	*F*(000) = 436
Triclinic, *P*1	*D*_x_ = 1.651 Mg m^−^^3^
Hall symbol: -P 1	Cu *K*α radiation, λ = 1.54184 Å
*a* = 7.1318 (10) Å	Cell parameters from 3632 reflections
*b* = 10.6239 (13) Å	θ = 4.3–73.8°
*c* = 11.9564 (13) Å	µ = 1.23 mm^−^^1^
α = 84.257 (10)°	*T* = 110 K
β = 74.497 (11)°	Chunk, pale yellow
γ = 77.559 (11)°	0.51 × 0.41 × 0.33 mm
*V* = 851.58 (18) Å^3^	

### Data collection

**Table:**

Oxford Xcalibur diffractometer with Ruby (Gemini Cu) detector	3344 independent reflections
Radiation source: Enhance (Cu) X-ray Source	2761 reflections with *I* > 2σ(*I*)
graphite	*R*_int_ = 0.021
Detector resolution: 10.5081 pixels mm^-1^	θ_max_ = 73.9°, θ_min_ = 4.3°
ω scans	*h* = −7→8
Absorption correction: multi-scan (*CrysAlis PRO*; Oxford Diffraction, 2009)	*k* = −13→13
*T*_min_ = 0.431, *T*_max_ = 1.000	*l* = −14→12
6248 measured reflections	

### Refinement

**Table:**

Refinement on *F*^2^	Primary atom site location: structure-invariant direct methods
Least-squares matrix: full	Secondary atom site location: difference Fourier map
*R*[*F*^2^ > 2σ(*F*^2^)] = 0.041	Hydrogen site location: inferred from neighbouring sites
*wR*(*F*^2^) = 0.118	H-atom parameters constrained
*S* = 1.07	*w* = 1/[σ^2^(*F*_o_^2^) + (0.0696*P*)^2^ + 0.2349*P*] where *P* = (*F*_o_^2^ + 2*F*_c_^2^)/3
3344 reflections	(Δ/σ)_max_ = 0.001
272 parameters	Δρ_max_ = 0.27 e Å^−^^3^
0 restraints	Δρ_min_ = −0.29 e Å^−^^3^

### Special details

**Table:**

Experimental. CrysAlisPro, Oxford Diffraction Ltd., Version 1.171.33.34d (release 27-02-2009 CrysAlis171 .NET) (compiled Feb 27 2009,15:38:38) Empirical absorption correction using spherical harmonics, implemented in SCALE3 ABSPACK scaling algorithm.
Geometry. All e.s.d.'s (except the e.s.d. in the dihedral angle between two l.s. planes) are estimated using the full covariance matrix. The cell e.s.d.'s are taken into account individually in the estimation of e.s.d.'s in distances, angles and torsion angles; correlations between e.s.d.'s in cell parameters are only used when they are defined by crystal symmetry. An approximate (isotropic) treatment of cell e.s.d.'s is used for estimating e.s.d.'s involving l.s. planes.
Refinement. Refinement of *F*^2^ against ALL reflections. The weighted *R*-factor *wR* and goodness of fit *S* are based on *F*^2^, conventional *R*-factors *R* are based on *F*, with *F* set to zero for negative *F*^2^. The threshold expression of *F*^2^ > σ(*F*^2^) is used only for calculating *R*-factors(gt) *etc*. and is not relevant to the choice of reflections for refinement. *R*-factors based on *F*^2^ are statistically about twice as large as those based on *F*, and *R*- factors based on ALL data will be even larger.

### Fractional atomic coordinates and isotropic or equivalent isotropic displacement parameters (Å^2^)

**Table:**

	*x*	*y*	*z*	*U*_iso_*/*U*_eq_	
O1A	0.6186 (2)	0.42327 (13)	1.18314 (12)	0.0358 (3)	
O2A	0.5417 (2)	0.62511 (13)	1.13392 (12)	0.0383 (3)	
N1A	0.6304 (2)	0.51540 (14)	1.11228 (13)	0.0261 (3)	
N2A	0.9515 (2)	0.68299 (13)	0.73199 (12)	0.0228 (3)	
H2AA	1.0452	0.7222	0.7376	0.027*	
N3A	0.7069 (2)	0.67430 (13)	0.64072 (12)	0.0236 (3)	
H3AB	0.6416	0.7048	0.5877	0.028*	
H3AC	0.6762	0.6076	0.6865	0.028*	
N4A	0.8981 (2)	0.82883 (13)	0.58308 (12)	0.0225 (3)	
H4AA	0.8336	0.8600	0.5299	0.027*	
H4AB	0.9943	0.8644	0.5909	0.027*	
C1A	1.0118 (2)	0.44934 (16)	0.77518 (15)	0.0229 (3)	
C2A	0.9141 (2)	0.57363 (15)	0.80935 (14)	0.0208 (3)	
C3A	0.7870 (2)	0.59582 (16)	0.91843 (14)	0.0221 (3)	
H3AA	0.7204	0.6807	0.9400	0.026*	
C4A	0.7597 (2)	0.49070 (16)	0.99526 (14)	0.0225 (3)	
C5A	0.8533 (2)	0.36602 (16)	0.96653 (15)	0.0246 (4)	
H5AA	0.8327	0.2956	1.0208	0.029*	
C6A	0.9788 (3)	0.34633 (16)	0.85597 (15)	0.0254 (4)	
H6AA	1.0437	0.2610	0.8347	0.030*	
C7A	1.1495 (3)	0.42731 (18)	0.65702 (16)	0.0298 (4)	
H7AA	1.0824	0.4706	0.5979	0.045*	
H7AB	1.1880	0.3345	0.6445	0.045*	
H7AC	1.2682	0.4624	0.6513	0.045*	
C8A	0.8505 (2)	0.72837 (15)	0.65149 (14)	0.0202 (3)	
O1B	0.59719 (17)	0.85654 (11)	0.47353 (10)	0.0254 (3)	
O21B	0.84884 (19)	1.00632 (12)	0.36978 (12)	0.0319 (3)	
O22B	0.7546 (2)	1.15265 (12)	0.24729 (12)	0.0324 (3)	
O41B	0.3034 (2)	1.09649 (12)	0.03718 (11)	0.0318 (3)	
O42B	0.1500 (3)	0.93647 (15)	0.08197 (15)	0.0518 (5)	
O61B	0.2382 (2)	0.64362 (13)	0.39470 (12)	0.0351 (3)	
O62B	0.3584 (2)	0.69013 (14)	0.52904 (12)	0.0353 (3)	
N2B	0.7376 (2)	1.05245 (13)	0.30647 (12)	0.0236 (3)	
N4B	0.2629 (2)	1.00057 (14)	0.09892 (13)	0.0292 (3)	
N6B	0.3236 (2)	0.71124 (14)	0.43296 (12)	0.0243 (3)	
C1B	0.5286 (2)	0.88544 (15)	0.38719 (14)	0.0206 (3)	
C2B	0.5836 (2)	0.98569 (15)	0.29955 (14)	0.0211 (3)	
C3B	0.4980 (2)	1.02404 (16)	0.20829 (14)	0.0224 (3)	
H3BA	0.5364	1.0924	0.1555	0.027*	
C4B	0.3543 (3)	0.96112 (16)	0.19447 (14)	0.0237 (3)	
C5B	0.3011 (2)	0.85874 (16)	0.26861 (14)	0.0230 (3)	
H5BA	0.2080	0.8137	0.2554	0.028*	
C6B	0.3831 (2)	0.82270 (15)	0.36097 (14)	0.0217 (3)	

### Atomic displacement parameters (Å^2^)

**Table:**

	*U*^11^	*U*^22^	*U*^33^	*U*^12^	*U*^13^	*U*^23^
O1A	0.0411 (8)	0.0400 (8)	0.0270 (7)	−0.0189 (6)	−0.0048 (6)	0.0092 (6)
O2A	0.0393 (8)	0.0368 (8)	0.0319 (7)	−0.0027 (6)	−0.0007 (6)	−0.0020 (6)
N1A	0.0239 (7)	0.0325 (8)	0.0248 (7)	−0.0119 (6)	−0.0070 (6)	0.0014 (6)
N2A	0.0240 (7)	0.0239 (7)	0.0247 (7)	−0.0118 (6)	−0.0099 (6)	0.0048 (6)
N3A	0.0255 (7)	0.0248 (7)	0.0243 (7)	−0.0104 (6)	−0.0114 (6)	0.0063 (5)
N4A	0.0227 (7)	0.0246 (7)	0.0239 (7)	−0.0101 (5)	−0.0096 (6)	0.0042 (5)
C1A	0.0220 (8)	0.0269 (8)	0.0236 (8)	−0.0084 (6)	−0.0096 (6)	−0.0005 (6)
C2A	0.0211 (8)	0.0240 (8)	0.0216 (8)	−0.0101 (6)	−0.0099 (6)	0.0036 (6)
C3A	0.0206 (8)	0.0227 (8)	0.0262 (8)	−0.0064 (6)	−0.0103 (7)	0.0003 (6)
C4A	0.0214 (8)	0.0284 (9)	0.0211 (8)	−0.0105 (6)	−0.0076 (6)	0.0013 (6)
C5A	0.0277 (9)	0.0238 (8)	0.0275 (9)	−0.0126 (7)	−0.0121 (7)	0.0048 (6)
C6A	0.0286 (9)	0.0212 (8)	0.0298 (9)	−0.0073 (7)	−0.0115 (7)	−0.0011 (7)
C7A	0.0312 (9)	0.0307 (9)	0.0271 (9)	−0.0075 (7)	−0.0054 (7)	−0.0024 (7)
C8A	0.0195 (8)	0.0207 (8)	0.0193 (7)	−0.0032 (6)	−0.0031 (6)	−0.0018 (6)
O1B	0.0296 (6)	0.0253 (6)	0.0264 (6)	−0.0099 (5)	−0.0138 (5)	0.0039 (5)
O21B	0.0288 (7)	0.0328 (7)	0.0420 (7)	−0.0141 (5)	−0.0201 (6)	0.0092 (6)
O22B	0.0406 (7)	0.0278 (7)	0.0374 (7)	−0.0190 (6)	−0.0184 (6)	0.0089 (5)
O41B	0.0458 (8)	0.0249 (6)	0.0293 (7)	−0.0084 (5)	−0.0183 (6)	0.0044 (5)
O42B	0.0762 (11)	0.0455 (9)	0.0594 (10)	−0.0350 (8)	−0.0505 (9)	0.0193 (8)
O61B	0.0454 (8)	0.0334 (7)	0.0345 (7)	−0.0234 (6)	−0.0129 (6)	0.0028 (5)
O62B	0.0369 (7)	0.0442 (8)	0.0326 (7)	−0.0218 (6)	−0.0176 (6)	0.0158 (6)
N2B	0.0251 (7)	0.0224 (7)	0.0253 (7)	−0.0087 (6)	−0.0072 (6)	0.0005 (5)
N4B	0.0382 (8)	0.0246 (8)	0.0308 (8)	−0.0088 (6)	−0.0179 (7)	0.0015 (6)
N6B	0.0218 (7)	0.0248 (7)	0.0266 (7)	−0.0076 (6)	−0.0054 (6)	0.0020 (6)
C1B	0.0201 (8)	0.0194 (7)	0.0227 (8)	−0.0032 (6)	−0.0060 (6)	−0.0024 (6)
C2B	0.0209 (8)	0.0203 (8)	0.0237 (8)	−0.0061 (6)	−0.0062 (6)	−0.0024 (6)
C3B	0.0253 (8)	0.0197 (8)	0.0223 (8)	−0.0052 (6)	−0.0056 (6)	−0.0015 (6)
C4B	0.0281 (9)	0.0242 (8)	0.0224 (8)	−0.0060 (7)	−0.0116 (7)	−0.0020 (6)
C5B	0.0227 (8)	0.0227 (8)	0.0259 (8)	−0.0066 (6)	−0.0075 (7)	−0.0034 (6)
C6B	0.0209 (8)	0.0213 (8)	0.0233 (8)	−0.0057 (6)	−0.0055 (6)	0.0001 (6)

### Geometric parameters (Å, °)

**Table:**

O1A—N1A	1.2306 (19)	C7A—H7AA	0.9800
O2A—N1A	1.217 (2)	C7A—H7AB	0.9800
N1A—C4A	1.468 (2)	C7A—H7AC	0.9800
N2A—C8A	1.344 (2)	O1B—C1B	1.241 (2)
N2A—C2A	1.4363 (19)	O21B—N2B	1.2334 (18)
N2A—H2AA	0.8800	O22B—N2B	1.2278 (18)
N3A—C8A	1.317 (2)	O41B—N4B	1.2364 (19)
N3A—H3AB	0.8800	O42B—N4B	1.227 (2)
N3A—H3AC	0.8800	O61B—N6B	1.2250 (19)
N4A—C8A	1.325 (2)	O62B—N6B	1.2280 (19)
N4A—H4AA	0.8800	N2B—C2B	1.4534 (19)
N4A—H4AB	0.8800	N4B—C4B	1.445 (2)
C1A—C2A	1.398 (2)	N6B—C6B	1.463 (2)
C1A—C6A	1.401 (2)	C1B—C2B	1.457 (2)
C1A—C7A	1.497 (2)	C1B—C6B	1.460 (2)
C2A—C3A	1.384 (2)	C2B—C3B	1.374 (2)
C3A—C4A	1.386 (2)	C3B—C4B	1.390 (2)
C3A—H3AA	0.9500	C3B—H3BA	0.9500
C4A—C5A	1.381 (2)	C4B—C5B	1.385 (2)
C5A—C6A	1.391 (2)	C5B—C6B	1.368 (2)
C5A—H5AA	0.9500	C5B—H5BA	0.9500
C6A—H6AA	0.9500		
			
O2A—N1A—O1A	123.73 (15)	C1A—C7A—H7AC	109.5
O2A—N1A—C4A	118.54 (14)	H7AA—C7A—H7AC	109.5
O1A—N1A—C4A	117.73 (15)	H7AB—C7A—H7AC	109.5
C8A—N2A—C2A	123.28 (13)	N3A—C8A—N4A	120.59 (14)
C8A—N2A—H2AA	118.4	N3A—C8A—N2A	120.75 (14)
C2A—N2A—H2AA	118.4	N4A—C8A—N2A	118.67 (14)
C8A—N3A—H3AB	120.0	O22B—N2B—O21B	122.00 (13)
C8A—N3A—H3AC	120.0	O22B—N2B—C2B	118.56 (13)
H3AB—N3A—H3AC	120.0	O21B—N2B—C2B	119.43 (13)
C8A—N4A—H4AA	120.0	O42B—N4B—O41B	122.85 (15)
C8A—N4A—H4AB	120.0	O42B—N4B—C4B	118.43 (14)
H4AA—N4A—H4AB	120.0	O41B—N4B—C4B	118.72 (14)
C2A—C1A—C6A	117.73 (16)	O61B—N6B—O62B	122.78 (14)
C2A—C1A—C7A	121.12 (15)	O61B—N6B—C6B	117.97 (14)
C6A—C1A—C7A	121.14 (16)	O62B—N6B—C6B	119.25 (13)
C3A—C2A—C1A	121.89 (14)	O1B—C1B—C2B	124.20 (14)
C3A—C2A—N2A	118.23 (15)	O1B—C1B—C6B	124.04 (15)
C1A—C2A—N2A	119.82 (15)	C2B—C1B—C6B	111.76 (14)
C2A—C3A—C4A	118.15 (15)	C3B—C2B—N2B	116.04 (14)
C2A—C3A—H3AA	120.9	C3B—C2B—C1B	124.33 (14)
C4A—C3A—H3AA	120.9	N2B—C2B—C1B	119.63 (14)
C5A—C4A—C3A	122.45 (16)	C2B—C3B—C4B	118.92 (15)
C5A—C4A—N1A	119.67 (14)	C2B—C3B—H3BA	120.5
C3A—C4A—N1A	117.86 (15)	C4B—C3B—H3BA	120.5
C4A—C5A—C6A	118.22 (15)	C5B—C4B—C3B	121.16 (15)
C4A—C5A—H5AA	120.9	C5B—C4B—N4B	119.33 (14)
C6A—C5A—H5AA	120.9	C3B—C4B—N4B	119.48 (14)
C5A—C6A—C1A	121.56 (16)	C6B—C5B—C4B	119.76 (15)
C5A—C6A—H6AA	119.2	C6B—C5B—H5BA	120.1
C1A—C6A—H6AA	119.2	C4B—C5B—H5BA	120.1
C1A—C7A—H7AA	109.5	C5B—C6B—C1B	123.85 (15)
C1A—C7A—H7AB	109.5	C5B—C6B—N6B	116.32 (14)
H7AA—C7A—H7AB	109.5	C1B—C6B—N6B	119.79 (14)
			
C6A—C1A—C2A—C3A	−0.7 (2)	O1B—C1B—C2B—C3B	−175.27 (16)
C7A—C1A—C2A—C3A	−179.70 (15)	C6B—C1B—C2B—C3B	4.9 (2)
C6A—C1A—C2A—N2A	176.60 (13)	O1B—C1B—C2B—N2B	4.3 (3)
C7A—C1A—C2A—N2A	−2.4 (2)	C6B—C1B—C2B—N2B	−175.56 (14)
C8A—N2A—C2A—C3A	−94.78 (19)	N2B—C2B—C3B—C4B	177.91 (15)
C8A—N2A—C2A—C1A	87.8 (2)	C1B—C2B—C3B—C4B	−2.5 (3)
C1A—C2A—C3A—C4A	0.8 (2)	C2B—C3B—C4B—C5B	−2.0 (3)
N2A—C2A—C3A—C4A	−176.51 (13)	C2B—C3B—C4B—N4B	179.95 (15)
C2A—C3A—C4A—C5A	−0.3 (2)	O42B—N4B—C4B—C5B	−4.7 (3)
C2A—C3A—C4A—N1A	177.78 (13)	O41B—N4B—C4B—C5B	176.06 (16)
O2A—N1A—C4A—C5A	−176.41 (15)	O42B—N4B—C4B—C3B	173.45 (18)
O1A—N1A—C4A—C5A	3.7 (2)	O41B—N4B—C4B—C3B	−5.8 (3)
O2A—N1A—C4A—C3A	5.4 (2)	C3B—C4B—C5B—C6B	3.5 (3)
O1A—N1A—C4A—C3A	−174.45 (14)	N4B—C4B—C5B—C6B	−178.40 (15)
C3A—C4A—C5A—C6A	−0.3 (2)	C4B—C5B—C6B—C1B	−0.7 (3)
N1A—C4A—C5A—C6A	−178.34 (14)	C4B—C5B—C6B—N6B	−178.21 (15)
C4A—C5A—C6A—C1A	0.4 (2)	O1B—C1B—C6B—C5B	176.90 (16)
C2A—C1A—C6A—C5A	0.1 (2)	C2B—C1B—C6B—C5B	−3.2 (2)
C7A—C1A—C6A—C5A	179.07 (15)	O1B—C1B—C6B—N6B	−5.6 (2)
C2A—N2A—C8A—N3A	1.0 (2)	C2B—C1B—C6B—N6B	174.21 (14)
C2A—N2A—C8A—N4A	−179.04 (15)	O61B—N6B—C6B—C5B	13.8 (2)
O22B—N2B—C2B—C3B	14.1 (2)	O62B—N6B—C6B—C5B	−165.22 (16)
O21B—N2B—C2B—C3B	−164.83 (15)	O61B—N6B—C6B—C1B	−163.84 (16)
O22B—N2B—C2B—C1B	−165.51 (15)	O62B—N6B—C6B—C1B	17.1 (2)
O21B—N2B—C2B—C1B	15.6 (2)		

### Hydrogen-bond geometry (Å, °)

**Table:**

*D*—H···*A*	*D*—H	H···*A*	*D*···*A*	*D*—H···*A*
N2A—H2AA···O22B^i^	0.88	2.20	3.0737 (18)	171
N3A—H3AB···O1B	0.88	2.03	2.7866 (18)	143
N3A—H3AB···O62B	0.88	2.35	3.0882 (18)	142
N3A—H3AC···O1A^ii^	0.88	2.32	2.9808 (19)	132
N4A—H4AA···O1B	0.88	1.98	2.7499 (18)	145
N4A—H4AA···O21B	0.88	2.34	3.0683 (19)	141
N4A—H4AB···O21B^i^	0.88	2.11	2.9572 (18)	163
C3A—H3AA···O41B^iii^	0.95	2.37	3.262 (2)	155
C7A—H7AB···O1B^iv^	0.98	2.56	3.447 (2)	151

Symmetry codes: (i) −*x*+2, −*y*+2, −*z*+1; (ii) −*x*+1, −*y*+1, −*z*+2; (iii) −*x*+1, −*y*+2, −*z*+1; (iv) −*x*+2, −*y*+1, −*z*+1.
